# Advances of nanopore direct sequencing technology and bioinformatics analysis for cell-free DNA detection and its clinical applications in cancer liquid biopsy

**DOI:** 10.3389/fmolb.2025.1662587

**Published:** 2025-10-15

**Authors:** Jiaxin Tan, Zetong Wu, Yunya Zhu, Biyuan Miao, Daofeng Xu, Jia Gu, Maohong Hu, Pingping Xu, Shaogui Wan

**Affiliations:** ^1^ Institute of Genomics and Precision Medicine, School of Medical Technology, Gannan Medical University, Ganzhou, China; ^2^ School of Basic Medicine, Gannan Medical University, Ganzhou, China; ^3^ Department of General Medicine, First Affiliated Hospital of Gannan Medical University, Ganzhou, China; ^4^ Department of Hepatobiliary Surgery, The First Affiliated Hospital of Gannan Medical University, Ganzhou, China; ^5^ Department of Data Science, City University of Macau, Macau, China; ^6^ Department of Colorectal Surgery, Zhongshan Hospital, Fudan University, Shanghai, China

**Keywords:** nanopore sequencing, cell-free DNA, methylation, copy number variations, liquid biopsy

## Abstract

Cell-free DNA (cfDNA) containing cancer information has become a key biomarker for cancer liquid biopsy. Current next-generation sequencing (NGS) technology for cfDNA detection often fail to capture multiomics information, such as fragmentomics, epigenetics, and genetics, in a single assay. Recently, Oxford Nanopore Technologies (ONT) has demonstrated advantages in acquiring cfDNA’s multiomics data by a single sequencing run. In this review, we summarize the recent advancements in library preparation and bioinformatic analyses for cfDNA methylation, copy number variations (CNVs), as well as other biomarkers derived from cfDNA sequencing on the ONT platform. Furthermore, we highlight the latest research progress in the clinical applications of multi-dimensional cfDNA features and outline the future directions of nanopore cfDNA sequencing. Overall, this review updates the understanding of cfDNA detection using nanopore sequencing, providing valuable insights for studies of cfDNA in cancer.

## 1 Background

According to the latest report by the International Agency for Research on Cancer (IARC), approximately 20 million new cancer cases and 9.7 million cancer-related deaths were recorded globally in 2022 (Bray et al., 2024). The incidence and mortality rates of cancer have been rising annually, making it one of the most prevalent and life-threatening diseases that severely impact human health and quality of life (Bray et al., 2024; Bray et al., 2021; Siegel et al., 2022; Sung et al., 2021). Therefore, early diagnosis and surveillance of cancer are critically important for cancer patient management. Tissue biopsies, as a traditional diagnosis method, face inherent limitations, including low patient acceptability due to procedural risks and the impracticality of repetitive sampling for assessing therapeutic efficacy (Heitzer et al., 2019; Li et al., 2021). Recently, liquid biopsy, as a minimally invasive or non-invasive approach, has exhibited many advantages in early cancer diagnosis and disease status monitoring. Among the liquid biopsy biomarkers, cell-free DNA (cfDNA) has been the most extensively utilized in clinical practice (Heitzer et al., 2019).

The current mechanisms for cfDNA release into bodily fluids primarily involve two pathways: the passive release through cellular apoptosis or necrosis and the active secretion via extracellular vesicles (Wan et al., 2017). In cancer patients, tumor cells also release cfDNA, known as circulating tumor DNA (ctDNA), into bodily fluids. The predominant fragment size of cfDNA is about 167 bp (Heitzer et al., 2013; Jiang et al., 2015), with a half-life ranging from 16 min to several hours in blood circulation (Diehl et al., 2008). During cancer initiation and progression, the multiomics information (including fragmentomics, epigenetics and genetics) of cfDNA in bodily fluids of healthy individuals exhibits significant alterations. These cfDNA molecules are associated with various clinical features, including tumor presence, tumor type, tumor size, tumor stage, and treatment response. Therefore, comprehensively and systematically unraveling the cfDNA profiles of cancer patients plays an essential role in cancer diagnosis, tumor type identification, and prognostic evaluation (Pascual et al., 2022; Liu et al., 2020; Shen et al., 2018).

Currently, next-generation sequencing (NGS) has been widely applied to cfDNA detection (Chen and Zhao, 2019). Systematic profiling of cfDNA typically necessitates conducting multiple experimental and sequencing runs via NGS. For instance, detecting cfDNA methylation features generally requires whole-genome bisulfite sequencing (WGBS), while analyzing cfDNA genetic features typically relies on whole-genome sequencing (WGS). Although NGS can accurately detect cfDNA epigenetic modifications and genetic changes through multiple sequencing runs, its short-read limitations inherently prevent the comprehensive acquisition of cfDNA fragment length features. In addition, PacBio sequencing (Yu et al., 2021), as a long-read single-molecule sequencing technology, seems to overcome the aforementioned limitations. However, the single-molecule real-time (SMRT) sequencing technology requires a substantial input of cfDNA, whereas the actual cfDNA content in the bodily fluids of cancer patients is typically low.This limitation may lead to reduced sequencing throughput and even failure to meet analytical demands. Notably, another third-generation sequencing (TGS) technology, Oxford Nanopore Technologies (ONT), enables simultaneous detection of multiomics features in cfDNA through a single sequencing run (Katsman et al., 2022). Additionally, ONT demonstrates significantly higher sequencing throughput when analyzing cfDNA compared to PacBio (Yu et al., 2023). With the advantages such as direct methylation detection, PCR-free amplification, long-read sequencing, high throughput, and short turnaround time, ONT exhibits unique strengths and promising clinical potential in the field of cfDNA analysis.

**FIGURE 1:**
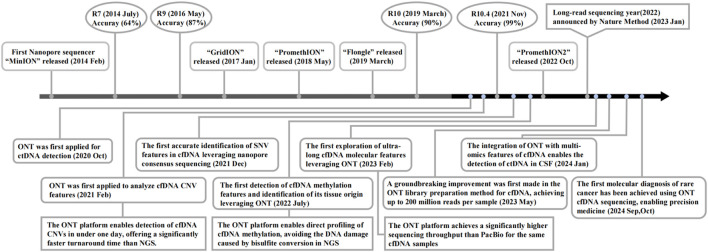
Timeline of nanopore sequencing progress and applications for cancer-derived cfDNA detection.

In recent years, nanopore sequencing technology has achieved continuous improvements in accuracy (Ashton et al., 2015; Minei et al., 2018; Karst et al., 2021; Srivathsan et al., 2024; Rang et al., 2018), leading to its increasingly widespread application in cfDNA detection and analysis, particularly demonstrating significant value in clinical oncology (Katsman et al., 2022; Bruzek et al., 2020; Martignano et al., 2021; Lau et al., 2023; Marcozzi et al., 2021) (Figure 1). Notably, Nature Methods proclaimed 2022 the “Year of Long-Read Sequencing,” authoritatively recognizing the burgeoning development of nanopore sequencing technology at that time. Tumor heterogeneity, as a key focus of clinical research, provides critical guidance for treatment strategy formulation (Zhu et al., 2026; Ahmed et al., 2025). Compared to traditional tissue biopsy, the cfDNA obtained through liquid biopsy encompasses all tumor lesions within a patient, enabling nanopore sequencing to potentially resolve tumor heterogeneity (Ahmed et al., 2022).

In this review, we provide a comprehensive overview of library preparation methods for cfDNA nanopore sequencing, followed by a summary of widely studied bioinformatic analysis methods for cfDNA methylation and copy number variations (CNVs) features. Furthermore, we delineate the clinical applications of cfDNA features detectable by nanopore sequencing technology in various cancers. Finally, we discuss the existing limitations and envision the potential future directions for cfDNA nanopore sequencing.

## 2 The library construction workflow of nanopore sequencing for cfDNA

**FIGURE 2:**
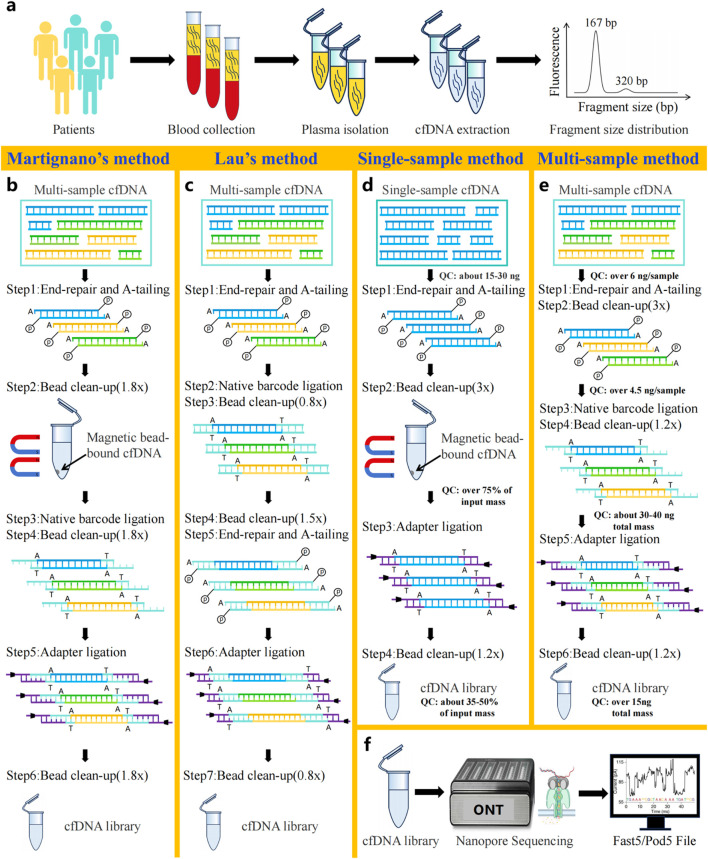
Workflow of cfDNA nanopore sequencing. **(a)** Patient blood collection and cfDNA extraction. **(b)** Library construction method for cfDNA developed by Martignano et al. **(c)** Library construction method for cfDNA developed by Lau et al. **(d)** Official single-sample cfDNA library preparation protocol for Oxford Nanopore sequencing. **(e)** Official multi-sample cfDNA library preparation protocol for Oxford Nanopore sequencing. **(f)** Principles of Oxford Nanopore sequencing technology.

Nanopore sequencing of cfDNA directly detects nucleic acid sequences and epigenetic features via measuring the characteristic current signals generated by single-stranded cfDNA molecules passing through the nanopores (Figure 2f) (Jain et al., 2015). Similar to Next-generation cfDNA sequencing, library construction also plays an essential role in Nanopore sequencing of cfDNA. And this process involves two key steps: extraction of cfDNA molecules from samples (Figure 2a) and ligation of sequencing adapters compatible with the nanopore platform. In order to develop suitable protocols for short-fragment cfDNA sequencing, several studies had improved genomic DNA (gDNA) library construction protocols before ONT released official protocols of cfDNA library preparation (Katsman et al., 2022; Yu et al., 2023; Martignano et al., 2021; Schmidt et al., 2024; Afflerbach et al., 2024; Van Der Pol et al., 2023).

Since the bead/sample ratio critically influences the size distribution of recovered DNA library fragments, recent studies have focused on optimizing it to increase the recovery efficiency of short cfDNA molecules (Katsman et al., 2022; Yu et al., 2023; Martignano et al., 2021; Schmidt et al., 2024; Afflerbach et al., 2024; Van Der Pol et al., 2023). For example, as demonstrated by Martignano et al., the application of cfDNA nanopore sequencing technology in lung cancer research led to the novel proposal of modifying solely the bead/sample ratio from 0.8× to 1.8× in all clean-up steps for the first time, without changing other suggestions of the EXP-NBD104 and SQK-LSK109 protocols (Figure 2b). Compared with the original protocol, it is surprising that this optimization protocol produced more sequencing reads (Martignano et al., 2021). Consequently, subsequent nanopore cfDNA sequencing studies have widely adopted this optimized ratio as a methodological benchmark (Katsman et al., 2022; Yu et al., 2023; Schmidt et al., 2024; Afflerbach et al., 2024; Van Der Pol et al., 2023). It is noteworthy that Yu et al. reported long cfDNA fragments longer than 500 bp had been detected in nanopore sequencing of maternal plasma cfDNA by Martignano’s method, which revealed the efficiency of long cfDNA fragments recovery using nanopores (Yu et al., 2023). Further, longer cfDNA fragments can provide more comprehensive epigenetic and CNVs information, resulting in more accurate clinical diagnosis (Van Der Pol et al., 2023).

In particular, Lau et al. developed a unique library preparation protocol specifically optimized for nanopore-based cfDNA sequencing. In this innovative approach, the researchers replaced the original ONT library construction reagents with alternative end-repair and ligation enzymes (Roche KAPA HyperPrep kit) and established a completely redesigned workflow (Figure 2c). Briefly, the workflow implemented barcode ligation directly after end-repaired cfDNA, omitting a clean-up step. Subsequently, two rounds of fragment clean-up were performed to remove impurities, preceding end repair and A-tailing to enable adapter ligation. It is worth noting that the structurally modified workflow boosts sequencing yield approximately 10-fold versus standard SQK-LSK109 with EXP-NBD196 workflow, even while reducing cfDNA input amount to 100 pg (Lau et al., 2023).

Currently, ONT has introduced two dedicated cfDNA library preparation workflows: the single-sample SQK-LSK114 and multiplexed SQK-NBD114.24 protocol (Figures 2d,e). Both methods incorporate essential steps, including end-repair, adapter ligation, and dual purification cycle. Of note, compared to the single-sample workflow, the multiplexed workflow reduces input amount by 60% per sample (6 ng vs. 15 ng) but necessitates additional barcode ligation to identify samples in pooled runs. Throughout the library preparation workflow, corresponding quality control standards were implemented at each key step to rigorously monitor experimental quality. Generally speaking, the two library preparation methods offer flexible solutions to accommodate diverse clinical research needs, supporting various sample types and throughput.

Additionally, Ridder’s team combined Rolling Circle Amplification (RCA) with nanopore sequencing for addressing the limitation of identifying single nucleotide variants (SNVs) in cfDNA at low sequencing depths, which significantly improves the detection sensitivity of low-frequency mutations in cfDNA by ONT (Marcozzi et al., 2021; Chen et al., 2025). However, there is a noticeable lack of amplification, which results in the loss of cfDNA epigenetic features. Hence, the approach is not suitable for cfDNA methylation analysis (Parras-Moltó et al., 2018).

## 3 Bioinformatics analysis based on various cfDNA features

As a representative of TGS technologies, one of the significant advantages of nanopore sequencing technology lies in its ability to reveal cfDNA profiles through a single sequencing run. It demonstrates prominent potential in applying cfDNA features, including fragmentomics, epigenetics, and genetics, to cancer diagnosis, tumor type identification, and treatment response monitoring. Among the diverse cfDNA features within cfDNA profiles (Table 1), Methylation Modification, CNVs, end motif, and fragment length can be directly detected by the ONT platform. The detection of cfDNA features such as chromosomal rearrangement and SNVs typically requires ONT combined with PCR technology or RCA. Currently, among the numerous cfDNA features, studies on cfDNA methylation and cfDNA CNVs are the most extensive. Therefore, we focus on summarizing the bioinformatic methods used to analyze the features of cfDNA methylation and CNVs.

**TABLE 1:** Comprehensive profiling of cfDNA using nanopore sequencing.

Cell-free DNA features	References
Methylation Modification	Katsman et al. (2022), Yu et al. (2023), Lau et al. (2023), Schmidt et al. (2024), Afflerbach et al. (2024), Sol et al. (2024)
Copy Number Variations	Katsman et al. (2022), Martignano et al. (2021), Schmidt et al. (2024); Afflerbach et al. (2024), Van Der Pol et al. (2023), Chen et al. (2025), Sol et al. (2024)
End Motif	Katsman et al. (2022), Yu et al. (2023)
Fragment Length	Katsman et al. (2022), Yu et al. (2023), Van Der Pol et al. (2023), Chen et al. (2025)
Single Nucleotide Variants	Bruzek et al. (2020), Marcozzi et al. (2021), Chen et al. (2025)
Chromosomal Rearrangement	Sampathi et al. (2022)

### 3.1 Methylation profiles of cfDNA

**FIGURE 3:**
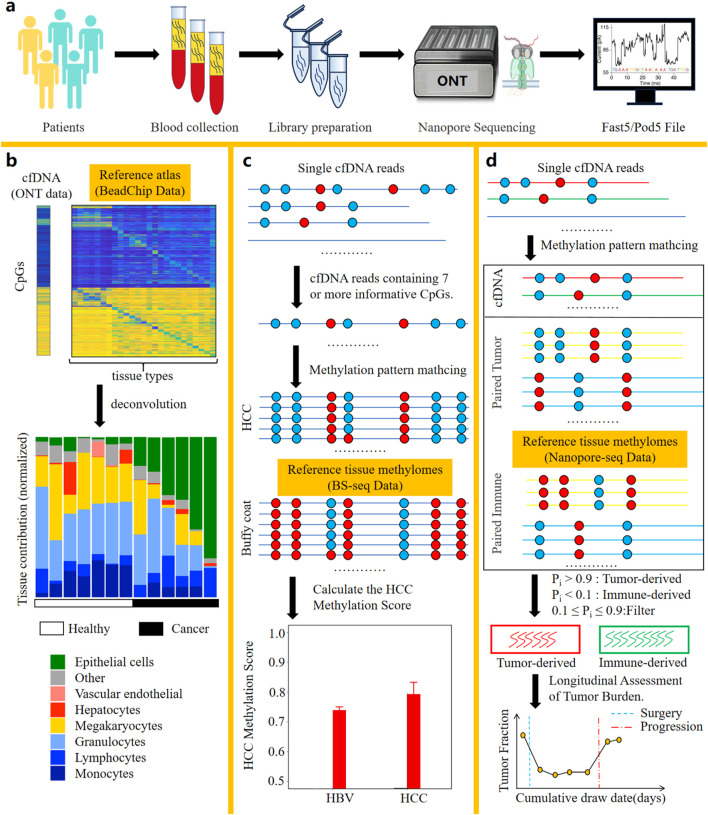
Bioinformatic analysis methods based on nanopore sequencing for identifying the tissue of origin of cfDNA. **(a)** Workflow of cfDNA nanopore sequencing. **(b)** The method by Katsman et al. Deconvolution of cfDNA samples using a reference methylation atlas from 25 healthy tissues enabled quantification of their tissue-specific contribution proportions. **(c)** The method by Yu et al. Comparative analysis of methylation patterns in long cfDNA fragments against reference profiles from HCC tissues and Buffy coat enabled single-molecule classification of cfDNA origin through probabilistic similarity scoring. Additionally, the HCC methylation score, calculated based on similarity scores of long cfDNA molecules, can be utilized as a diagnostic indicator for hepatocellular carcinoma. **(d)** The method by Lau et al. Comparative analysis of methylation patterns in all cfDNA fragments against reference profiles from gastrointestinal tumor tissues and PBMCs enabled single-molecule classification of cfDNA origin through probabilistic similarity scoring. The number of tumor-derived cfDNA molecules can be used to evaluate the patient’s tumor burden and monitor the patient’s response to treatment. Crucially, this method requires integrated cfDNA, tumor tissue, and PBMC samples derived from the same patient.

DNA methylation represents one of the most prevalent types of epigenetic modifications. Aberrant alterations in DNA methylation status are closely associated with diseases, especially with the development and progression of cancer. In the field of oncology, determining the tissue origin of cfDNA is of great significance. And this information plays a critical role in cancers of unknown primary (CUP) and early cancer diagnosis. Furthermore, identifying the tissue origin of cfDNA can uncover drug-induced secondary tissue damage (e.g., toxic effects on healthy tissues), which is an essential consideration in antitumor drug development and treatment response monitoring. Recent studies have demonstrated that cfDNA molecules harboring tissue-specific methylation sites can be leveraged to identify cell death in specific tissues (Loyfer et al., 2023; Akirav et al., 2011; Lebastchi et al., 2013; Lehmann-Werman et al., 2016; Moss et al., 2018). Collectively, these findings provide a theoretical foundation for tracing the tissue origin of cfDNA molecules through methylation signatures. In recent years, due to the ability of nanopore sequencing to directly detect methylation modifications in cfDNA (Figure 3a) without requiring bisulfite conversion, methods leveraging nanopore sequencing to identify the tissue origins of cfDNA have continuously emerged (Katsman et al., 2022; Yu et al., 2023; Lau et al., 2023). Table 2 summarizes the methods using nanopore sequencing to detect the tissue origins of cfDNA.

**TABLE 2:** Nanopore sequencing-based method for tissue of origin tracing of cfDNA.

Characteristics	Katsman’s method	Yu’s method	Lau’s method
Tissue-of-origin	25 tissues	2 tissues (HCC, Buffy coat)	2 tissues (Colorectal, PBMCs)
Reference atlas	BeadChip Based	WGBS Based	ONT Based
Paired tumor tissues	Free	Free	Based
Principle	Deconvolution (NNLS algorithm)	Comparing methylation pattern	Comparing methylation pattern
Single molecule tracing	No	Yes	Yes
Output	Proportion of tissue contribution	HCC methylation score	Tumor Burden
Practical application	Accurately classify 6 lung cancer samples and 7 healthy samples. (Accuracy:100%)	Accurately classify 6 HBV samples and 8 HCC samples. (Accuracy:85.7%)	Successfully longitudinally assessed the tumor burden in three gastrointestinal cancer patients
Minimum coverage	0.2X	Not evaluated	Not evaluated

WGBS, Whole-Genome Bisulfite Sequencing; ONT, oxford nanopore technologies; NNLS, Non-Negative Least Squares; HCC, hepatocellular carcinoma; PBMCs, Peripheral Blood Mononuclear Cells; HBV, hepatitis B virus carriers.

Katsman et al. performed nanopore sequencing on cfDNA samples from six lung cancer donors and seven healthy donors (Katsman et al., 2022). After obtaining the electrical signal files from cfDNA samples, methylation information was called and annotated with Illumina HumanMethylation450 BeadChip probe IDs. This ensures compatibility between the nanopore-derived methylation data and reference methylation atlas data generated from the BeadChip platform. Leveraging a BeadChip-generated reference methylation atlas of 25 healthy human tissues (Moss et al., 2018), deconvolution analysis was applied to the cfDNA sample to determine the percentage contribution from each of the 25 tissues to the cfDNA composition (Figure 3b). Notably, Loyfer et al. recently published a more comprehensive reference methylation atlas (Loyfer et al., 2023), which is based on WGBS technology. This atlas expands the reference methylation profiling to encompass the entire genome and increases the number of reference tissue types to 39. The fragment-level deconvolution method developed by them, compared to CpG-site-level deconvolution methods, achieves an order-of-magnitude improvement in the accuracy of cfDNA tissue-of-origin tracing. These valuable data and algorithmic resources can be integrated with nanopore sequencing, further advancing the practical clinical applications of nanopore cfDNA sequencing.

Yu et al. performed nanopore sequencing on cfDNA samples from 8 hepatocellular carcinoma (HCC) patients and 6 hepatitis B virus (HBV) patients. After obtaining the electrical signal files from the cfDNA samples, the methylation information of the cfDNA samples was called. Subsequently, reads were filtered to retain those containing 7 or more informative CpGs (Yu et al., 2023) for single-molecule tissue-of-origin tracing analysis. The reference methylation atlas used in this method is derived from WGBS data published by Chan et al. (Chan et al., 2013), with a sequencing depth of 36X for HCC gDNA and 75X for Buffy Coat gDNA. Informative CpGs are defined as those with a methylation level difference (Δβ) ≥0.3 between Buffy Coat and HCC. The methylation patterns of reads containing 7 or more informative CpGs are compared against the reference methylation atlas. The similarity score of each read to the methylation atlas of HCC and Buffy Coat tissues is quantified and denoted as S(HCC) and S(BC), respectively. If the similarity score calculation for a read results in S(HCC) > S(BC), the read is classified as originating from HCC; otherwise, it is classified as originating from Buffy Coat. Validation shows that the accuracy of this tracing method for cfDNA classification reaches 86%. Finally, by synthesizing the S(HCC) and S(BC) scores of each read, the HCC Methylation Score is calculated, which can be used to evaluate whether a patient has HCC (Figure 3c).

Lau et al. established a reference methylation atlas, with an average sequencing depth of 28X across the reference atlas, utilizing nanopore sequencing of gDNA derived from primary tumor tissue and PBMCs from 3 gastrointestinal cancer patients (Lau et al., 2023). After establishing the reference methylation atlas, nanopore sequencing was performed on cfDNA samples from three gastrointestinal cancer patients. Following the acquisition of electrical signal files from the cfDNA samples, methylation information was computationally called. The methylation patterns of all reads are compared against the reference methylation atlas to obtain the probability of each read originating from the primary tumor tissue (f_i_^tumor^) and PBMCs (f_i_^immune^). These probabilities are then normalized to calculate the tumor score P_i_ for each read as P_i_ = f_i_^tumor^/(f_i_^tumor^ + f_i_^immune^). A dual-threshold system is employed to classify each read: reads with P_i_ > 0.9 are classified as originating from tumor tissue, those with P_i_ < 0.1 are classified as originating from PBMCs, and reads with 0.1 ≤ Pi ≤ 0.9 cannot be definitively classified as either tumor-derived or PBMCs-derived. After quantifying the number of tumor-derived cfDNA molecules, this metric can be used to calculate the tumor burden of the sample and enable longitudinal monitoring of tumor progression and treatment response in patients (Figure 3d).

It is noteworthy that although the analysis of tissue origin using cfDNA has yielded promising results, current research predominantly focuses on identifying ctDNA admixed within the bulk cfDNA (derived from healthy cells), while there remains a lack of methods leveraging ctDNA to explore tumor heterogeneity. CtDNA originates from all deceased tumor cells across various tumor foci in the human body, and distinct subpopulations of tumor cells exhibit significant heterogeneity in their epigenetic features (Ahmed et al., 2022) (especially methylation patterns), which makes it possible to develop methods for identifying individual tumor heterogeneity using cfDNA nanopore sequencing technology (Schmidt et al., 2024; Sol et al., 2024). Exploring tumor heterogeneity holds promise for elucidating mechanisms of drug resistance (Chatterjee and Bivona, 2019), identifying tumor subtypes (Fischer et al., 2025), and developing more personalized treatment regimens for patients, thereby advancing precision medicine.

### 3.2 CNVs profiles of cfDNA

**FIGURE 4:**
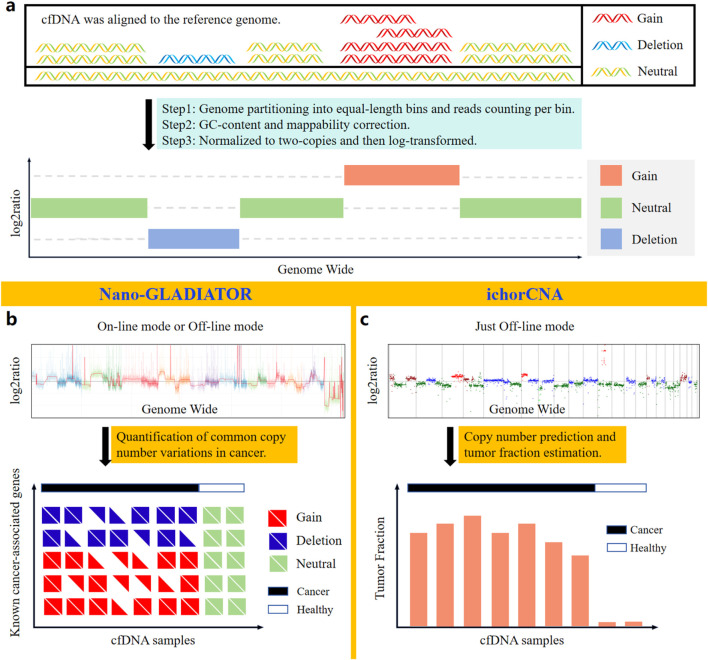
Bioinformatic analysis methods based on nanopore sequencing for identifying CNVs in cfDNA. **(a)** Principles of CNVs analysis. **(b)** Nano-GLADIATOR can detect CNVs in cancer-related genes and has an online analysis mode that operates concurrently with sequencing. **(c)** ichorCNA enables detection of cancer-associated CNVs and quantification of tumor fraction in cfDNA samples.

CNVs refer to genomic alterations involving DNA segments of at least 50 bp (Alkan et al., 2011). These alterations can be present at variable copy numbers in comparison to the reference genome (Figure 4a). CNVs serve as a hallmark of various cancers, and specific CNVs can define tumor types and progression stages, thus being critically linked to clinical diagnosis and prognostic evaluation (Hieronymus et al., 2018).

Current sequencing-based CNVs detection predominantly employs the read count (RC) method (Adalsteinsson et al., 2017; Magi et al., 2019), which identifies CNVs regions by statistically analyzing genome-wide RC (Figure 4a). Traditional NGS short-read sequencing relies on PCR amplification for library preparation, thereby introducing significant GC bias. Additionally, the short reads from NGS exhibit pronounced mappability bias. In contrast, long-read ONT sequencing, which is PCR-free and capable of spanning tandem repeat regions and complex genomic regions with its long reads, avoids the impacts of GC bias and mappability bias on RC statistics. Due to the aforementioned advantages, nanopore sequencing technology achieves higher sensitivity and specificity than NGS in detecting CNVs (Magi et al., 2016). Notably, due to the length distribution of cfDNA reads, which typically peaks around 167 bp, the sequenced cfDNA reads are generally short. As a result, nanopore sequencing data of cfDNA also exhibit certain mappability biases.

Currently, the software tools (Table 3) based on nanopore sequencing technology for detecting cfDNA CNVs include NanoGLADIATOR (Magi et al., 2019) and ichorCNA (Adalsteinsson et al., 2017). They first divide the genome into consecutive and non-overlapping bins and then calculate the RC within each bin. After calculating the RC for each bin, the RC values are corrected for GC bias and mappability bias. The corrected RC values are normalized to two-copies and subsequently log-transformed to generate log_2_ratio values. Genomic regions with elevated log_2_ratio values exhibit copy number gains, whereas regions with reduced log_2_ratio values indicate copy number losses (Figure 4a).

**TABLE 3:** Nanopore sequencing-based cfDNA copy number variations detection methods.

Characteristics	Nano-GLADIATOR	ichorCNA
Function	Identify CNVs	Identify CNVs and TF estimation
Applicable platform	NGS and TGS	NGS and TGS
Mode	On-line mode and Off-line mode	Off-line mode
Applicable data type	(gDNA and cfDNA) WGS data	(gDNA and cfDNA) WGS data
Minimum coverage	2M reads (∼0.1X)	2M reads (∼0.1X)

CNVs, Copy Number Variations; TF, tumor fraction; NGS, Next-Generation Sequencing; TGS, Third-Generation Sequencing; gDNA, genomic DNA; cfDNA, Cell-free DNA; WGS, Whole-Genome Sequencing.

Figures 4b,c display the CNVs profiles generated by NanoGLADIATOR and ichorCNA, respectively. NanoGLADIATOR’s online mode supports real-time generation of CNVs profiles during sequencing runs. These profiles enable the identification of common cancer-associated gene CNVs. In contrast, despite ichorCNA lacking an online mode, it not only detects CNVs but also incorporates an additional tumor fraction (TF) estimation function, enabling the estimation of the TF in individuals. Notably, the analytical requirements for both NanoGLADIATOR and ichorCNA can be met with approximately 2 million reads (Katsman et al., 2022; Martignano et al., 2021), which corresponds to a sequencing depth of approximately 0.1X. Bioinformatic methods adapted for low-depth sequencing data inherently reduce sequencing time and computational resource demands. The development of such bioinformatic analysis methods reduces the barriers to clinically implementing nanopore sequencing to detect CNVs in cancer patients, particularly in resource-constrained healthcare settings.

Although tools such as NanoGLADIATOR and ichorCNA can accurately identify CNVs/TF in low-depth cfDNA nanopore sequencing data, these tools often fail to correctly resolve samples with extremely low tumor fractions. Meanwhile, due to the inherently low sequencing depth and lack of allelic information in the cfDNA nanopore sequencing approach, these tools often struggle to reliably and explicitly distinguish large numbers of subclones. In the future, these issues may be addressed through advancements in cfDNA nanopore sequencing library preparation methods or the development of novel analytical approaches.

### 3.3 Other feature profiles of cfDNA

Cancer fragmentomics is an emerging field that primarily investigates the differences between ctDNA and normal cfDNA (Im et al., 2021; Underhill et al., 2016; Mouliere et al., 2011). In fragmentomics, the most fundamental cfDNA features are the end motif and fragment length. A recent study discovered that the CCCA motif exhibited significant differences between healthy individuals and cancer patients by analyzing the proportions of all end motifs with the t-test (Katsman et al., 2022). In another study by Yu et al. distinguished HCC from non-HCC samples via plotting the receiver operating characteristic (ROC) curve based on cfDNA end motif information (Choy et al., 2022). In addition, since ctDNA fragments are typically ∼10 bp shorter than normal cfDNA (Mouliere et al., 2018; Udomruk et al., 2021), Chen et al. integrated cfDNA fragment length features with Non-negative Matrix Factorization (NMF) (Lee and Seung, 1999; Renaud et al., 2022) to calculate the TF based on cfDNA fragment length distribution, achieving robust classification between healthy and cancer samples (Chen et al., 2025). Overall, the current study of fragmentation analysis methods for nanopore cfDNA sequencing is in an early stage, and it is essential to develop more specialized bioinformatic methods for elucidating the intricate fragmentomics of cfDNA.

In addition to those cfDNA features mentioned above that can be directly detected by the ONT platform, SNVs and chromosomal rearrangement of cfDNA can also be accurately detected by combining RCA or PCR technology with nanopore sequencing and appropriate bioinformatic analysis methods. Marcozzi et al. successfully detected point mutations in the TP53 gene via calculating the Fraction Mutation method, which counted the ratio of the number of reads with detected point mutations to the number of non-mutated reads in the TP53 gene based on RCA-nanopore cfDNA sequencing (Marcozzi et al., 2021). In another study, Chen et al. first performed WGS on tumor gDNA from all patients and used Strelka (Saunders et al., 2012) to identify all tumor-informed somatic SNVs. In this study, the proportion of reads with SNVs in paired cfDNA samples was calculated to estimate the TF using prior information from tumor somatic mutations (Chen et al., 2025). Using this method, the TF effectively distinguished cancer patients from healthy individuals and accurately predicted the treatment response in a patient with granulosa cell tumor of the ovary. In addition to RCA-based methods, PCR-based targeted nanopore sequencing can also accurately detect SNVs. By leveraging prior knowledge of cancer-specific gene mutations, targeted sequencing of relevant genes can be performed with ultra-high sequencing depth, mitigating the impact of the high base-calling error rate inherent in ONT. This approach significantly improves the accuracy of Variant Allele Fraction (VAF) calculations. A recent study combined ONT and PCR to precisely calculate the VAF of multiple genes in pediatric high-grade glioma patients and used these multi-gene VAF profiles to predict therapeutic response (Bruzek et al., 2020). However, all methods for detecting SNVs in cfDNA require prior knowledge of cancer-associated mutations. For cancer types where such prior mutation information is unavailable, SNV-based detection approaches are not applicable. Furthermore, beyond epigenetic information, research teams have now begun to explore B-ALL heterogeneity using immunology-related prior knowledge and have achieved promising results. For detecting chromosomal rearrangements in cfDNA, Sampathi et al. employed PCR to amplify B-cell-specific rearrangements of the immunoglobulin heavy chain (IGH) region, followed by nanopore sequencing of the amplified products (Sampathi et al., 2022). After sequence alignment, the software Feature Counts was used to quantify various immunoglobulin heavy variable (IGHV) sequences. Quantitative analysis of IGHV sequences in cfDNA enables the detection of clonal heterogeneity and dynamic tracking of individual B-Cell Acute Lymphoblastic Leukemia (B-ALL) clone responses throughout the treatment course. Collectively, nanopore cfDNA sequencing enables accurate detection of cfDNA features, including SNVs and chromosomal rearrangements. These bioinformatic approaches demonstrate promising potential for cancer diagnosis and therapeutic response monitoring.

## 4 Clinical applications of cfDNA based on nanopore sequencing

It is well known that both methylation and CNVs of cfDNA can serve as promising cancer-associated biomarkers (Liu et al., 2020; Klein et al., 2021; Lenaerts et al., 2019). Moreover, nanopore sequencing technology, with its unique advantages of long-read native DNA sequencing, has been shown to play an important role in cfDNA-based cancer liquid biopsy. Here, we summarize the advances of cfDNA in cancer liquid biopsy utilizing nanopore sequencing technology (Table 4).

**TABLE 4:** Summary clinical applications of nanopore cfDNA sequencing.

Cancer	Patients	Sample	Library construction	Features of cfDNA	Clinical significance	References
Lung cancer	6 lung cancer patients cases, 4 healthy controls	Plasma	Martignano’s method, direct sequencing	CNVs	Diagnosis, guiding therapeutic strategy, dynamic prognostic evaluation	Martignano et al. (2021)
Lung cancer	6 lung adenocarcinoma cases, 7 healthy controls	Plasma	Martignano’s method, direct sequencing	Methylation, CNVs, nucleosome position, fragment length, end motif	Diagnosis, monitoring recurrence, dynamic prognostic evaluation	Katsman et al. (2022)
Lung cancer, bladder cancer	22 lung cancer cases, 3 healthy controls, 8 bladder cancer cases, 2 non-cancer controls	Plasma, urine	Refer to Martignano’s method, PCR-based sequencing	CNVs, nucleosome position, fragment length	Diagnosis, monitoring recurrence, dynamic prognostic evaluation	Van Der Pol et al. (2023)
Brain cancers	99 brain cancer patients	CSF	Refer to Martignano’s method, direct sequencing	Methylation, CNVs	Diagnosis, dynamic prognostic evaluation, monitoring recurrence	Afflerbach et al. (2024)
Brain cancer	1 Glioblastoma patient	CSF	—	Methylation, CNVs	Diagnosis	Sol et al. (2024)
Brain cancer	12 pHGG cases, 6 healthy controls	CSF	The SQK-LSK109 method, PCR-based sequencing	SNVs	Diagnosis, guiding therapeutic strategy	Bruzek et al. (2020)
Lymphoma	1 intravascular B-cell lymphoma patient	CSF	Refer to Martignano’s method, direct sequencing	Methylation, CNVs	Diagnosis, guiding therapeutic strategy	Schmidt et al. (2024)
Gastrointestinal cancer	23 CRC cases,1 metastatic CRC case, 1 metastatic pNEC case, 1 metastatic CCA case, 5 healthy controls	Plasma	Lau’s method, direct sequencing	Methylation	Diagnosis, guiding therapeutic strategy, monitoring recurrence	Lau et al. (2023)
Liver cancer	8 HCC cases, 6 HBV carriers controls	Plasma	Refer to Martignano’s method, direct sequencing	Methylation, fragment length, end motif	Diagnosis	Yu et al. (2023)
EAC, OVCA, GCT	5 metastatic EAC cases, 2 recurrent adult-type GCT of the ovary cases, 7 OVCA cases, 7 healthy controls	Plasma, ascites	NanoRCS, RCA-based sequencing	CNVs, SNVs, fragment length	Diagnosis, guiding therapeutic strategy, monitoring recurrence	Chen et al. (2025)
HNSCC	3 HPV-negative HNSCC patients	Plasma	CyclomicsSeq, RCA-based sequencing	SNVs	Diagnosis, monitoring recurrence	Marcozzi et al. (2021)
ALL	5 B-ALL patients	Plasma, CSF, BMMCs	The SQK-PBK004 method, PCR-based sequencing	VDJ-rearrangements	Diagnosis, guiding therapeutic strategy, dynamic prognostic evaluation	Sampathi et al. (2022)

EAC, esophageal adenocarcinoma; OVCA, ovarian cancer; GCT, granulosa cell tumor; HNSCC, head and neck squamous cell carcinoma; HPV-negative HNSCC, human papillomavirus-negative head and neck squamous cell carcinoma; ALL, acute lymphoblastic leukemia; B-ALL, B-cell acute lymphoblastic leukemia; pHGG, pediatric high-grade glioma; HCC, hepatocellular carcinoma; HBV, hepatitis B virus carriers; CSF, cerebrospinal fluid; cfDNA, cell-free DNA; PCR-based, polymerase chain reaction-based; NanoRCS, Nanopore Rolling Circle Amplification-enhanced Consensus Sequencing; RCA, rolling circle amplification; CNVs, copy number variations; SNVs, single nucleotide variants; VDJ-rearrangements, variable, diversity, and joining gene segment rearrangements; CRC, colorectal cancer; pNEC, pancreatic neuroendocrine carcinoma; CCA, cholangiocarcinoma; BMMCs, bone marrow mononuclear cell.

### 4.1 Lung cancer

Lung cancer, as the most prevalent cancer worldwide, poses a significant threat to human health and represents the leading cause of cancer-related mortality globally (Sung et al., 2021; Kratzer et al., 2024). Substantially, some studies have demonstrated that both early diagnosis and precision treatment can improve 5-year survival rates in lung cancer patients (Kratzer et al., 2024; Wu et al., 2020). In this context, several studies devoted to using the latest nanopore cfDNA sequencing technology for the diagnosis and precision treatment of lung cancer recently (Katsman et al., 2022; Martignano et al., 2021; Van Der Pol et al., 2023). For instance, Katsman and his colleagues carried out a comparative study that sequenced the methylation of plasma cfDNA from both healthy individuals and lung cancer patients using the ONT platform. Specifically, they demonstrated that nanopore sequencing could reliably detect cell-of-origin and cancer-specific cfDNA methylation features via Illumina-based cfDNA methylation datasets previously published (Moss et al., 2018; Fox-Fisher et al., 2021; Zhou et al., 2018; Nguyen et al., 2021), combining with their sequencing data for cell type deconvolution analysis. Additionally, they also substantiated that global DNA hypomethylation can serve as a universal biomarker for ctDNA, enabling effective discrimination between lung cancer patients and healthy controls (Katsman et al., 2022).

Furthermore, ONT-based CNVs analysis of cfDNA from lung cancer patients can provide an important molecular typing basis for precision treatment decision-making, demonstrating an important clinical application value (Nguyen et al., 2021; Nukaga et al., 2017; Sakre et al., 2017; Rihawi et al., 2019). For example, Martignano et al. pioneered the application of Nanopore sequencing to profile plasma cfDNA CNVs in lung cancer patients. Several CNVs in cancer-relevant genes, which had been found to be associated with drug resistance in lung cancer in previous studies (Nukaga et al., 2017; Sakre et al., 2017; Rihawi et al., 2019), were been accurately identified by this study (Martignano et al., 2021). In addition, several studies have consistently conducted a comparative analysis of CNVs detection in ctDNA from lung cancer patients using both Nanopore sequencing and Illumina sequencing. These studies found that Nanopore sequencing could achieve comparable accuracy to conventional short-read sequencing platforms in detecting CNVs from plasma-derived cfDNA (Katsman et al., 2022; Martignano et al., 2021; Van Der Pol et al., 2023).

Overall, these findings indicate that nanopore-based CNVs profiling of cfDNA can serve as a robust molecular tool for guiding targeted therapy selection and advancing precision medicine in lung cancer.

### 4.2 Brain cancers

The highly heterogeneous characteristic of central nervous system (CNS) cancers (Louis et al., 2021; Ahmed et al., 2025; Hervey-Jumper and Berger, 2016), combined with the anatomical constraints of certain lesions (Safaee et al., 2014; Chen et al., 2020), fundamentally limits the utility of traditional tissue biopsy in clinical diagnosis. In contrast, the liquid biopsy technology using nanopore sequencing for cfDNA, with its noninvasive and reproducible features (Chabon et al., 2020), presents a promising alternative for cancer diagnosis, longitudinal monitoring, and recurrence assessment in brain cancers. For instance, Afflerbach et al. demonstrated this potential by performing comprehensive methylome and CNVs analyses of cerebrospinal fluid (CSF)-derived cfDNA from patients with 20 different brain cancer types using nanopore sequencing. Notably, both methylation and CNVs analyses demonstrated the capability to detect ctDNA even in samples without known residual lesions. While nanopore cfDNA CNVs analysis detected ctDNA in 88% of positive samples, methylation profiling crucially identified the remaining 12%, underscoring the necessity of multimodal liquid biopsy approaches. Collectively, these findings underline that integrative analysis of nanopore sequencing-derived cfDNA methylation and CNVs signatures enhances detection sensitivity, thereby facilitating preliminary brain cancer diagnosis. Furthermore, the identification of disease stage-specific CNVs profiles in cfDNA supports the clinical potential of longitudinal CNVs monitoring for recurrence surveillance and therapy response evaluation (Afflerbach et al., 2024).

Subsequently, Sol et al. and Schmidt et al. successfully diagnosed a primary molluscum contagiosum glioblastoma and an intravascular large B-cell lymphoma, respectively, by combining methylation and CNVs analysis of CSF cfDNA using nanopore sequencing technology (Schmidt et al., 2024; Sol et al., 2024). These studies not only demonstrate the essential role of CSF cfDNA methylation and CNVs analysis in detecting radiologically occult CNS neoplasms but also overcome the diagnostic limitations of traditional biopsy for cerebral involvement in lymphoma diagnosis, offering a promising approach for brain cancers.

In addition, Bruzek et al. also successfully diagnosed pediatric high-grade glioma (pHGG) by detecting cancer-associated mutations in CSF-derived cfDNA. Their approach, which combined targeted PCR amplification with nanopore sequencing, enabled accurate brain cancer diagnosis (Bruzek et al., 2020).

Hence, these studies provide compelling evidence that nanopore sequencing of cfDNA mutations can serve as a reliable diagnostic approach for brain cancers.

### 4.3 Other cancers

Given the high sensitivity and specificity of cfDNA methylation in cancer detection (Chabon et al., 2020), nanopore sequencing has been increasingly adopted for tissue-of-origin analysis of cfDNA methylation in extra-pulmonary and non-CNS cancers (Yu et al., 2023; Lau et al., 2023). For example, nanopore sequencing of long plasma cfDNA enabled Yu et al. to achieve methylation-based discrimination between patients with HCC and HBV carriers through tissue-of-origin analysis (Yu et al., 2023). Moreover, Lau et al.'s nanopore-based methylation atlas of colorectal cancer (CRC) tissues and peripheral blood mononuclear cells (PBMCs) facilitated ctDNA detection via single-molecule classification, with subsequent longitudinal studies confirming that ctDNA levels reliably tracked radiographic changes in gastrointestinal cancer (Lau et al., 2023). Collectively, these results demonstrate the promising clinical utility of nanopore-based cfDNA methylation profiling in cancer diagnosis and personalized treatment guidance.

Beyond cfDNA methylation profiling, the identification of cancer-derived somatic mutations in ctDNA constitutes an important strategy for cancer biomarker development (Logsdon et al., 2020). As a result, a study by Marcozzi and colleagues established that RCA-coupled nanopore sequencing achieved a detection limit of 0.02% VAF for TP53 mutations in ctDNA, permitting serial assessment of cancer dynamics through mutation quantification in head and neck squamous cell carcinoma (HNSCC) (Marcozzi et al., 2021). The findings demonstrate that this approach enables sensitive detection of minimal residual disease (MRD), thus significantly improving both recurrence monitoring and prognostic evaluation.

Critically, nanopore sequencing achieves simultaneous detection of epigenomic, genetic, and fragmentomic features in a single assay (Wang et al., 2021; Capper et al., 2018), significantly enhancing diagnostic precision in oncology. Chen et al. developed an innovative Nanopore RCA-enhanced Consensus Sequencing (NanoRCS) technology to comprehensively detect CNVs, SNVs, and fragmentomics of cfDNA in esophageal adenocarcinoma (EAC), ovarian cancer (OVCA), and granulosa cell tumor (GCT). The result revealed that while single-modality approaches exhibited limited sensitivity for low-TF samples, multimodal integration substantially improved TF detection accuracy, providing compelling theoretical support for implementing multi-omics strategies in cancer diagnostics (Chen et al., 2025).

## 5 Prospective and future direction

As the latest cfDNA detection methodology, nanopore sequencing enables the comprehensive revelation of cfDNA profiles through a single sequencing run. Its capacity to harness multi-omics features demonstrates immense potential in cancer diagnosis, tumor type identification, and prognostic evaluation for patients. Furthermore, nanopore sequencing also addresses the long turnaround time limitation of previous NGS. With the shortest turnaround time of several hours, it supports obtaining analytical reports of patient cfDNA on the day of detection (Martignano et al., 2021; Van Der Pol et al., 2023), which provides strong support for the practical clinical application of cfDNA nanopore sequencing technology.

Although nanopore sequencing holds promising clinical application prospects, there are currently critical challenges that urgently need to be addressed. In scenarios where the concentration of cfDNA in patient plasma or other bodily fluids is extremely low, nanopore sequencing may fail to generate a sufficient number of reads to meet analytical demands (Afflerbach et al., 2024). Although nanopore sequencing technology can be combined with PCR to leverage limited cfDNA for generating sufficient reads, this approach introduces the side effect of eliminating epigenetic modification information and also introducing significant GC bias. Thus, improving the extraction efficiency of cfDNA or developing more efficient cfDNA library preparation workflows may be effective methods to generate sufficient reads.

Improving sequencing throughput on the experimental end can provide more cfDNA information from patients, however, it is equally essential to develop analytical methods that are capable of effectively detecting tumor-related signals in cfDNA under low sequencing depth on the analytical end. Currently, the limitation of low-concentration cfDNA leads to an average sequencing depth typically below 1×. Such low sequencing depth implies that certain tumor-associated signals present in cfDNA may not be accurately captured, making it more prone to generating false-positive results (Katsman et al., 2022). Furthermore, although analytical methods in some fields were not specifically designed for cfDNA—as their original purpose was to detect tumor gDNA (Schmidt et al., 2024; Afflerbach et al., 2024; Sol et al., 2024), (Vermeulen et al., 2023; Kuschel et al., 2023), [80]—this misalignment results in a significant decrease in diagnostic efficacy when these methods are applied to cfDNA detection (Afflerbach et al., 2024). Consequently, developing bioinformatic analysis methods adapted to the low sequencing depth of cfDNA remains a critical challenge to be addressed for the clinical application of cfDNA nanopore sequencing technology.

Recently, to maximize the capability of nanopore sequencing in revealing cfDNA profiles through a single sequencing run, some studies have sought to integrate multi-omics features of cfDNA to enhance the accuracy of cancer diagnosis and treatment response monitoring (Afflerbach et al., 2024; Chen et al., 2025). By integrating multidimensional features of cfDNA, more comprehensive and complementary information can be obtained, thereby improving the accuracy of cancer diagnosis and treatment response monitoring in patients. Despite the fact that nanopore sequencing can directly detect multiomics features of cfDNA, its current limitation is the high basecalling error rates, which necessitates reliance on RCA for identifying features such as SNVs. This limitation not only increases the workload in library preparation but also compromises epigenetic modification information in cfDNA, further reducing the amount of usable cfDNA information obtainable in a single sequencing run. Hence, improving the accuracy of nanopore basecalling remains a critical challenge to be addressed for the practical clinical application of cfDNA nanopore sequencing technology.

Whether enhancing the performance of nanopore sequencing in analyzing cfDNA at the experimental or analytical end, such improvements must ultimately be grounded in clinical applications. To enable clinicians to rapidly and conveniently access patient cfDNA analytical reports, it is essential to establish a standardized operating procedure (SOP) spanning from blood collection to the generation of cfDNA reports. On the experimental side, it is imperative to standardize sample handling and library preparation protocols. On the analytical side, developing a comprehensive bioinformatic analysis pipeline for cfDNA profiles is essential. As ONT becomes more widely adopted for cfDNA detection, the gradually maturing SOP for cfDNA nanopore sequencing will enhance the interpretation of tumor-related information, driving this technology toward broader clinical applications in the future.

## Glossary

cfDNA: Cell-free DNA
ONT: Oxford nanopore technologies
CNVs: Copy number variations
IARC: International agency for research on cancer
ctDNA: Circulating tumor DNA
NGS: Next-generation sequencing
WGBS: Whole-genome bisulfite sequencing
WGS: Whole-genome sequencing
SMRT: Single-molecule real-time
TGS: Third-generation sequencing
gDNA: genomic DNA
RCA: Rolling circle amplification
SNVs: Single nucleotide variants
CUP: Cancers of unknown primary
HCC: Hepatocellular carcinoma
HBV: Hepatitis B virus
RC: Read count
TF: Tumor fraction
ROC: Receiver operating characteristic
NMF: Non-negative matrix factorization
VAF: Variant allele fraction
IGH: Immunoglobulin heavy chain
IGHV: Immunoglobulin heavy variable
B-ALL: B-Cell acute lymphoblastic leukemia
CNS: Central nervous system
CSF: Cerebrospinal fluid
pHGG: Pediatric high-grade glioma
CRC: Colorectal cancer
PBMCs: Peripheral blood mononuclear cells
HNSCC: Head and neck squamous cell carcinoma
MRD: Minimal residual disease
NanoRCS: Nanopore RCA-enhanced consensus sequencing
EAC: Esophageal adenocarcinoma
OVCA: Ovarian cancer
GCT: Granulosa cell tumor
SOP: Standardized operating procedure
